# Analysis of Variability of Functionals of Recombinant Protein Production Trajectories Based on Limited Data

**DOI:** 10.3390/ijms23147628

**Published:** 2022-07-10

**Authors:** Shuting Liao, Kantharakorn Macharoen, Karen A. McDonald, Somen Nandi, Debashis Paul

**Affiliations:** 1 Graduate Group in Biostatistics, University of California, Davis, CA 95616, USA; stliao@ucdavis.edu; 2 Department of Chemical Engineering, University of California, Davis, CA 95616, USA; kmacharoen@ucdavis.edu (K.M.); kamcdonald@ucdavis.edu (K.A.M.); 3 Global HealthShare, University of California, Davis, CA 95616, USA; 4 Department of Statistics, University of California, Davis, CA 95616, USA

**Keywords:** production trajectories, limited data, ANOVA, linear constraints, simultaneous hypothesis tests, resampling techniques

## Abstract

Making statistical inference on quantities defining various characteristics of a temporally measured biochemical process and analyzing its variability across different experimental conditions is a core challenge in various branches of science. This problem is particularly difficult when the amount of data that can be collected is limited in terms of both the number of replicates and the number of time points per process trajectory. We propose a method for analyzing the variability of smooth functionals of the growth or production trajectories associated with such processes across different experimental conditions. Our modeling approach is based on a spline representation of the mean trajectories. We also develop a bootstrap-based inference procedure for the parameters while accounting for possible multiple comparisons. This methodology is applied to study two types of quantities—the “time to harvest” and “maximal productivity”—in the context of an experiment on the production of recombinant proteins. We complement the findings with extensive numerical experiments comparing the effectiveness of different types of bootstrap procedures for various tests of hypotheses. These numerical experiments convincingly demonstrate that the proposed method yields reliable inference on complex characteristics of the processes even in a data-limited environment where more traditional methods for statistical inference are typically not reliable.

## 1. Introduction

Many biological experiments involve production of certain recombinant molecule over a period of time under different experimental conditions. Thus, the data associated with such experiments are inherently longitudinal. One long-standing problem is to compare these optimum trajectories across different factors or experimental conditions (or treatments), which is a core topic of longitudinal data analysis [1,2,3,4]. In most of these studies, the object of interest is typically the expected amount of the ingredient being measured, and one has multiple replicates to accommodate a comprehensive ANOVA (Analysis of Variance) approach to deal with the problem of ascribing effects of various factors.

Indeed, the traditional approach to such inferential questions has been through the application of repeated measures designs [5,6,7]. However, in many real-life lab based biological experiments, one key constraint is the number of data points or replicates that can be obtained, due to the time, costs and resources associated with completing each condition, particularly in scaling-up experiments.

Furthermore, in many instances, as we discuss below, the key object of interest is not the level of the target molecule itself but some, possibly nonlinear, functionals of the production trajectory. For instance, this functional could be (a) the time it takes for the accumulation of the target molecule to reach a prespecified value (to be referred to as the “optimal time-to-harvest”); (b) the maximum production level (=the maximum of the production trajectory); or (c) the maximum productivity, defined as the maximum of the amount divided by time over the duration of the experiment.

### 1.1. Scientific Context

As a good example, butyrylcholinesterase (BChE) circulating in human blood plasma is a tetrameric hydrolase enzyme that can be potentially used as a prophylactic and/or therapeutic factor against organophosphorus nerve agent poisoning [8]. However, the use of purified BChE from human blood plasma in clinical stages is limited by its cost, which is estimated to be $20,000 per 400 mg dose [9]. Thus, recombinant human BChE (rBChE) has been developed in several host expression systems, including transgenic rice cell suspension cultures, to be used as an alternative source of BChE.

Our lab developed metabolically-regulated transgenic rice cell suspensions under the rice alpha amylase 3D (RAmy3D) promoter to produce rice-made recombinant human BChE (rrBChE) [10,11]. In nature, the RAmy3D promoter in rice cells derived from rice seed, is suppressed in a sugar-rich environment but activated in a sugar-starved environment [12,13,14]. In other words, the RAmy3D promoter-based transgenic rice cell suspensions are grown in a sugar-rich medium for production and transferred into sugar-free medium for rrBChE production.

Biological experiments of the kind described above are both time-consuming and expensive. A major challenge of growing plant cell suspension cultures is the slow growth rate of plant cells compared to microbial and mammalian cells. For example, it takes 6–7 days for transgenic rice cells to reach mid-to-late exponential growth phase, followed by the medium exchange to replace spent growth medium with sugar-free medium, and another 4–5 days post-induction for rrBChE expression [10,11]. In other words, the cultivation time of transgenic rice cell suspensions in a batch culture is 10–12 days.

When it comes to an experiment with several factors or conditions, the number of bioreactor replicates is likely to be restricted due to time of cultivation and limited equipment. Therefore, there might be difficulties when interpreting the data or choosing the optimal sets of conditions. Given the cost, the information delivered by the data is crucial, and thus we would like to understand the data in a more comprehensive way by developing statistical methods. For instance, it will be interesting and meaningful to characterize the production curves over time, particularly when the measurement time points are limited in practice.

We are able to predict production quantities at any experimental time points, in addition to those at observed time points. Furthermore, statistical inference will be useful to measure and indicate the factors’ impact on the difference among multiple experimental conditions in terms of certain metrics. Thus, in the aforementioned rrBChE study, we would like to provide a robust and effective statistical approach as a validated way to interpret the data better and address experimental questions through a statistical framework. For example, we can use inference procedures to determine and compare varying metrics, such as the “optimal time-to-harvest” of each factor based on certain levels of statistical significance, which will indicate the effect of factors behind the limited data.

Therefore, in this study, we employ novel statistical approaches to tackle limited data to build trajectory models using previously reported bioreactor data [11] to predict outcomes of interest, such as the “optimal time-to-harvest”, maximum rrBChE production level and maximum productivity. In addition, estimating the trajectory of the production level is a part of Quality by Design (QbD) [15] that is essential in biomanufacturing where a computationally feasible statistical method is involved in modeling based on available data.

### 1.2. Statistical Challenges and Contributions

Analysis of the variability of functionals of protein production trajectories across different experimental conditions presents several novel statistical challenges. One key requirement is to ensure that the underlying production trajectories are monotonic (i.e., either increasing or decreasing functions of time), without which some of the quantities of interest are not even properly defined. At the same time, due to the limited number of data points at which these trajectories are typically measured, it is imperative to borrow information across trajectories in order to ensure that we have sufficient degrees of freedom left for comparing the parameters across the factors.

Another challenge is that, due to both the limited number of data points and the restrictions imposed by the monotonicity of production trajectories, any statistical inference procedure that directly relies on large sample theory will have limited accuracy or may be misleading. Moreover, since some of the parameters (functionals of the production trajectories) or process metrics of interest are nonlinear, the standard ANOVA framework that relies on the linear model theory does not apply.

In this paper, we primarily focus on comparing the equality of the parameters by means of simultaneous pairwise comparisons, which can be formulated as a multiple hypothesis testing problem. Thus, the key statistical challenge is to develop a methodology that (a) ensures monotonicity of the fitted trajectories and (b) can handle simultaneous inference for arbitrary functionals of the trajectories with a limited amount of data.

In order to address these challenges, we adopt the following three-pronged approach. First, following the ideas in [16], we model the production trajectories by representing them in a B-spline basis and incorporate the monotonicity constraint by imposing linear inequality constraints on the B-spline coefficients. We fit the trajectories by using a constrained least squares regression procedure that is implemented through a quadratic programming approach. Next, for statistical inference on the parameters of interest, we use *bootstrap*, or resampling procedures. We compare the efficacies of several different versions of bootstrap, namely, the residual bootstrap, parametric bootstrap and nonparametric bootstrap.

Finally, since we conduct simultaneous inference involving many pairwise comparisons, we adopt a method for imparting control on the *false discovery rate* (i.e., the fraction of false detections) while constructing the simultaneous confidence intervals involving many parameters, using a technique developed in [17]. In summary, we provide a comprehensive framework for simultaneous statistical inference on several process metrics that are functionals of biochemical production (or growth) trajectories, based on fairly limited amounts of data, with empirical validity.

### 1.3. Goals of the Study

For the biological experimental study, the goal is to develop an effective and efficient system that is able to scale-up the production of rrBChE given the costs and limited resources. From the statistical side, one of the goals of this study is to analyze the variability of production trajectories for a limited data set of the recombinant protein production by using rrBChE as a model study.

Another goal is to use statistical approaches to determine the optimal time to harvest a recombinant protein during a protein production process. A further goal is to compare across different bootstrap procedures for their relative effectiveness in terms of statistical inference when the data are limited. The last goal is achieved through an extensive numerical simulation study mimicking the recombinant protein production experiments.

## 2. Results

### 2.1. Simulation Study

In this subsection, we present a simulation study illustrating the effectiveness of the proposed bootstrap-based inference procedures and accuracy of the corresponding confidence intervals. This numerical simulation also allows us to make a comparison among the different bootstrap procedures.

**Settings:** We assume the number of factors I=3, and the time interval is (0,9] with number of time points J=9. All factors share the same time points {1,2,3,4,5,6,7,8,9}. We assume there are L=5 basis functions for cubic B-splines with equally spaced knots. For each time point, we have n=5 replicates. Suppose $Y_{ijk}=\mu_{i}\left(t_{ij}\right)+\epsilon_{ijk}$, where the noise level is $\sigma_{j}^{2}\in \left(1,3.5\right)$. Our main interest is the “optimal time-to-harvest” $\theta_{i}$.

**Figure 1:**
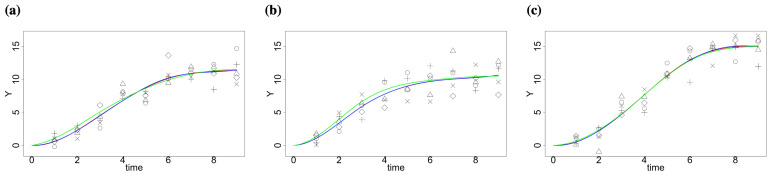
Simulated data and estimation by the standard least square procedure and quadratic programming framework for different factors; true data are denoted by different types of points; the true curve μ(t) is marked in green; fitted curves with linear constraints are in red, while the fitted curves by standard least square procedure are in blue (**a**) Factor 1, (**b**) Factor 2 and (**c**) Factor 3.

The simulated data and estimation by the standard least square procedure (without constraints) and quadratic programming framework (with constraints) are shown in Figure 1. Though the estimations using two methods are similar in our case, there is a difference. The estimated curve by the least square procedure for factor 3 shows a decreasing pattern at the last time point, while the one with constraints remains non-decreasing (Figure S1).

**Figure 2:**
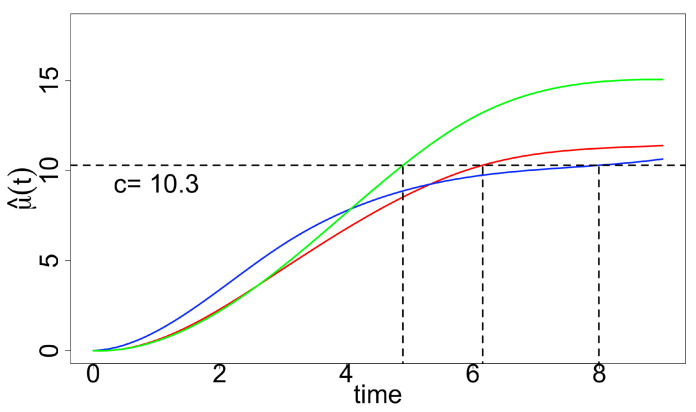
Fitted curves estimated with constraints (factors 1–3 are in red, blue and green, respectively); ${\hat{\theta }}_{i}$ given by a specified level (c=10.3) is indicated by vertical lines.

Since we are interested in estimating the growth curve and “optimal time-to-harvest”, how we fit the data matters. In addition, using the standard least square procedure may result in oscillations in estimation. The “optimal time-to-harvest” parameter $\theta_{i}$ is of interest with a pre-specified level c=10.3 (Figure 2), and we use the residual (nonparametric) bootstrap method for inference. We compute both percentile bootstrap confidence intervals and bias-corrected and accelerated bootstrap interval ($BC_{a}$) since the bootstrap sampling distributions involving $\theta_{2}$ are skewed (Table 1). Both types of intervals suggest that $\theta_{1}$ and $\theta_{2}$ are significantly different from $\theta_{3}$; however, we can see that $BC_{a}$ is slightly better than the general ones in terms of the length of intervals.

### 2.2. Analysis of rrBChE Data

**Figure 3:**
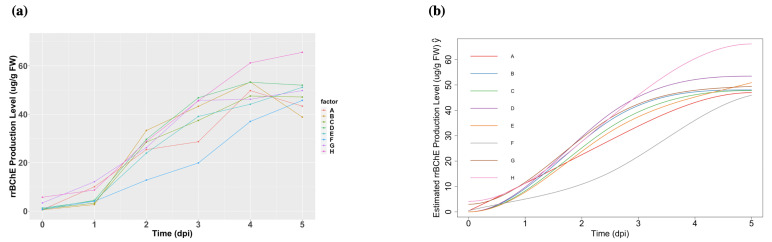
(**a**): The observed cell-associated rrBChE production levels in factors A to H. (**b**): Fitted curves with monotonicity in factors A to H constraints.

The rrBChE data is available at Dryad [18]. We have I=8 factors labeled A to H and primarily focus on the protein production level (ug rrBChE/g FW rice cells) after sugar induction. We have J=6 time points (days post-induction, dpi). Although one potential issue is that we only have one replicate (n=1) at each time for each factor, our framework is able to handle this and make appropriate inferences. The observed data and estimations are shown in Figure 3.

#### 2.2.1. “Optimal Time-to-Harvest” $\theta_{i}$ and “Optimum Stopping Time” $\gamma_{i}$

**Figure 4:**
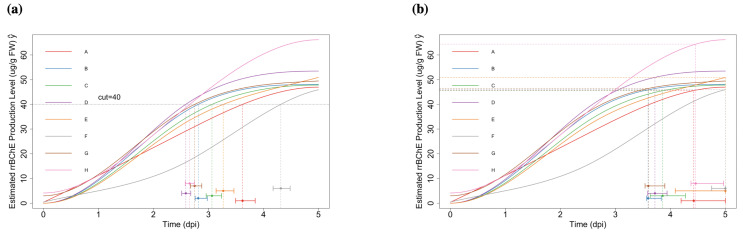
(**a**): Fitted curves with specified level (cut-off c=40 (μg/g FW)); bootstrap confidence intervals of $\theta_{i}$ are also shown (using *t*-distributed noise). (**b**): Fitted curves with specified level ($\tilde{c}=0.1$); bootstrap confidence intervals of $\gamma_{i}$ are also shown (using *t*-distributed noise).

With the pre-specified level c=40 (μg/g FW), we obtain the estimates ${\hat{\theta }}_{i}$ and make inferences by using the parametric bootstrap method (see Figure 4a). For the “optimum stopping time” $\gamma_{i}$, we set the level $\tilde{c}=0.1$ and repeat the procedures (see Figure 4b). We compute the corresponding confidence intervals by using normal noise and *t*-distributed noise separately. We found that the results were not sensitive to the type of noise (normal or *t*-distributed) that we are used. More details about the related confidence intervals can be found in Tables S5 and S6 in the Supplementary Materials.

#### 2.2.2. Simultaneous Inference—Maximum Production Level $\tau_{i}$

The hypotheses are:

$$\begin{array}{c} H_{0}:\tau_{i}=\tau_{i^{\prime }}\;\;\mathrm{vs}.\;\;H_{a}:\tau_{i}\neq \tau_{i^{\prime }} \end{array}$$

where $\tau_{i}=\max_{t}\mu_{i}\left(t\right)$ for i=1,⋯,I. We use the Benjamini–Hochberg procedure for FDR control. Since the null hypothesis enables computation of estimates using the quadratic programming framework, we use both the nonparametric (residual) bootstrap and parametric bootstrap (using *t*-distributed noise) to compute two versions of *p*-values. We found that that the rankings of *p*-values by the two different variants of bootstrap procedures were highly positively correlated, which means our method is robust and not sensitive to the way we compute *p*-values.

All indicated significant pairs by the residual bootstrap and percentile bootstrap CI method are shown in Table 2 and Table 3. The results related to using the null bootstrap distribution to compute *p*-values are shown in the Supplementary Materials (Tables S1 and S2). It is interesting to see that two versions of *p*-values indicate similar results from residual bootstrap and parametric bootstrap, respectively. However, the nonparametric (residual) bootstrap leads to a more conservative conclusion (seven to eight significant pairs), compared to the parametric one (13 significant pairs).

#### 2.2.3. Simultaneous Inference—Maximum “Unweighted” Productivity $\psi_{i}$

We now demonstrate that a wider variety of applications can be tackled by the proposed framework. For example, we can make inferences related to the maximum “unweighted” productivity. We consider using the maximum “unweighted” productivity

(1) $$\begin{array}{c} \psi_{i}:=\max_{t}h_{i}^{u}\left(t\right) \end{array}$$

as the quantity of interest, where

(2) $$\begin{array}{c} h_{i}^{u}\left(t\right):=\frac{\mu_{i}\left(t\right)}{\left(t+T_{i}^{\left(c\right)}\right)} \end{array}$$

$T_{i}^{\left(c\right)}$ is the days of cultivation before the induction.

**Figure 5:**
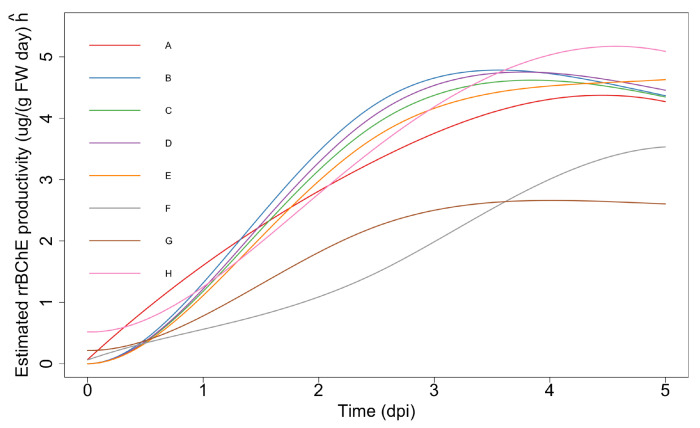
Estimated “unweighted” productivity curves based on observed data. Note that $T^{\left(c\right)}=\left(6,6,6,7,6,8,14,8\right)$.

The estimated “unweighted” productivity curves are shown in Figure 5. In this case, since the parameter is a nonlinear function of the trajectories, it is not possible to obtain the *p*-values using the null bootstrap distribution option. Instead, we use the correspondence between hypothesis testing and finding confidence intervals.

We apply the same procedures that we used for the maximum production level to the maximum “unweighted” productivity, using both the parametric bootstrap and residual bootstrap methods. Tables S3 and S4 (in the Supplementary Material) show that residual bootstrap method leads to more conservative results (14 significant pairs) compared to the parametric one (21 significant pairs).

### 2.3. Summary of Findings

#### 2.3.1. Comparison between Two Versions of *p*-Values

The results of the two bootstrap methods imply that the rankings of two versions of *p*-values are highly positively correlated (≥0.96), which means that our method is robust and not sensitive to the way in which we compute *p*-values.

#### 2.3.2. Comparison of Residual Bootstrap and Parametric Bootstrap

**Figure 6:**
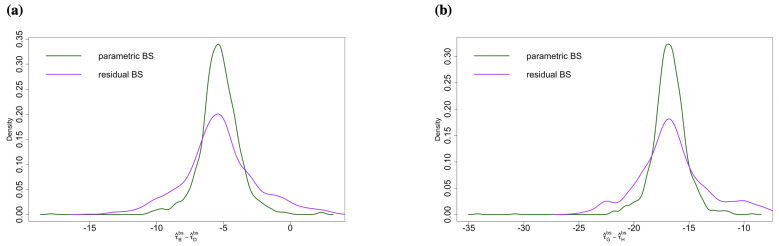
Comparison: empiricalparametric bootstrap sampling distribution and empirical residual bootstrap sampling distribution of (**a**) ${\hat{\tau }}_{B}^{bs}-{\hat{\tau }}_{D}^{bs}$, which is significant in the parametric bootstrap case but not in the residual bootstrap case. (**b**) ${\hat{\tau }}_{G}^{bs}-{\hat{\tau }}_{H}^{bs}$, which is significant in both cases.

Tables S1 and S2 may be compared for a performance of different bootstrap strategies. First, within each table, we can see the *p*-values by using the null and duality of *p*-value and CI are consistent based on the rank and its correlation. Second, we can see that residual bootstrap is more conservative (Table S1 only has seven significant differences), and the *p*-values are larger. We can see the main difference depends on the variability of data (the way we generate bootstrap samples). Parametric bootstrap samples show higher variability (we make use of the distribution assumption), while residual ones have lower variability. This means that the residual bootstrap is more conservative, and the result is consistent with Figure 6.

Compared to the parametric bootstrap method, the residual bootstrap method is more conservative (see Tables S1 and S2). Comparing the parametric and residual bootstrap sampling distributions for parameters (Figure 6), it is clear that the residual bootstrap method leads to more widely spread sampling distributions, which yields larger *p*-values and fewer significant testing results. Again, the residual bootstrap method is not particularly effective when the number of factors is small. I=8 might not be sufficient, and a choice needs to be made to make inferences.

## 3. Methods and Materials

### 3.1. Data Collection Method

A 5 L bioreactor (BioFlo 3000, formerly New Brunswick Scientific, Eppendorf Inc., Hauppauge, NY, USA) was used to study the production of rrBChE under eight different conditions as previously described [11] and summarized in Table 4. In brief, the effects of dissolved oxygen (DO) were conducted in factors (runs) A–E using a two-stage batch culture (the medium exchange was performed to replace spent sugar-rich medium with sugar-free medium to induce the promoter).

Factors F–H were operated in single-stage batch culture (no medium exchange; production was simply induced by sugar depletion from cellular uptake) with or without controlling DO and using 50% of the usual initial sucrose concentration during the growth phase. For each factor, samples were taken every day during days 0–5 post induction (dpi) to quantify the rrBChE activity in the cell extract and culture medium using a modified Ellman assay [19] and assuming a specific activity of 260 U/mg crude rrBChE to convert the activity to the rrBChE production level (μg/g fresh weight of rice cells) [11].

### 3.2. Modeling Production Trajectories

There were I≥2 factors (or treatments), and each treatment was applied to several independently chosen experimental units (bioreactors). Further, the response (e.g., the rrBChE concentration in the bioreactor) was measured at observation times $0<t_{i1}<\cdots <t_{iJ}=T$, say, for J≥2 (this allows the observation times to be different for different factors). Let us denote the mean response curve (at time t≥0) corresponding to the *i*-th factor as $\mu_{i}\left(\cdot \right)$. We assumed that $\mu_{i}\left(\cdot \right)$ is a monotonically increasing function of time, over the observation time window [0,T].

For simplicity as well as statistical efficiency, we assumed a *balanced experimental design*—that is, the sample size at each observational time was the same for every treatment. The measurement process is destructive, and thus, for any particular experimental unit, we only have one measurement, at the time of sampling the bioreactor. Therefore, to obtain reasonably accurate measurement for the whole trajectory, we required replicates (i.e., multiple experimental units) for each time $t_{ij}$ and each treatment *i*.

Let *n* denote the number of replicates assigned to each combination (i,j), which corresponds to a balanced design. Note that we allow n=1 since, in practice, only limited data are available particularly in certain biological experiments. We denote the response from the *k*-th experimental unit, in the *i*-th factor group, measured at time $t_{ij}$, to be $Y_{ijk}$.

(3) $$Y_{ijk}=\mu_{i}\left(t_{ij}\right)+\epsilon_{ijk},\;j=1,\cdots ,J;k=1,\cdots ,n;i=1,\cdots ,I.$$

where $\epsilon_{ijk}$ are independent random variables with mean 0 and unknown, common variance $\sigma^{2}>0$. In practice, we may allow the number of time points *J* to depend on index *i* as well.

We used a basis representation approach for modeling the mean trajectories $\mu_{i}$. In particular, we used cubic B-spline basis functions [20] for representing the functions. For each *i*, assuming *L* cubic B-spline basis functions are used to $\mu_{i}\left(t\right)$, we can write

(4) $$\begin{array}{c} \mu_{i}\left(t\right)=\Sigma_{l=1}^{L}\alpha_{il}B_{l}\left(t\right),\;i=1,\cdots ,I \end{array}$$

where $\left\{\alpha_{il}\right\}$ are the basis coefficients. The number *L* of basis functions used to model the growth/production trajectories is a user-specified positive integer that controls the degree of complexity of the trajectories, with larger values allowing for more complex shapes. In practice, *L* may be determined by utilizing data from pilot studies through a cross-validated linear regression procedure, which involves setting aside a random subset of the data (referred to as “validation data”) and using it to compare the prediction errors of the fitted trajectories corresponding to different values of *L* based on the “training data”.

A great advantage of the spline representation is that, since the functions $B_{l}\left(\cdot \right)$ are non-negative, the curve $\mu_{i}$ is nonnegative provided the coefficients $\alpha_{il}$ are so. A more significant advantage, from the point of view of modeling “production curves” of the type considered here, is that the condition that $\mu_{i}\left(t\right)$ is non-decreasing in *t* can be imposed by simply requiring that

(5) $$\begin{array}{c} \alpha_{i(l+1)}\geq \alpha_{il}\;\mathrm{for}\;l=1,\cdots ,L-1 \end{array}$$

for all *i* [16,21]. This is a reasonable assumption for batch production of a recombinant protein if the protein is stable in the culture medium (e.g., there is no consumption and/or degradation of the product but simply accumulation due to production). Note that this model is a simplified version of a more general ANOVA framework that enables quantifying possible interactions between the treatments and time. In Section 4, we discuss this possible extension to a two-factor ANOVA with potential interactions, and the associated linear constraints.

If we ignore the inequality constraints (5), the linear model given by (3), (4) can be fitted through an ordinary least squares procedure. The resulting estimate of $\mu_{i}\left(t\right)$ for any *t* will be *unbiased* (i.e., the average of the estimates across all possible samples equals the true value of the parameter) and will have an approximate Gaussian distribution for reasonably large values of *n*. In this case, we can rely on the large sample theory for statistical inference on the parameters of interest.

However, in the applications considered here, we need to consider the monotonicity constraints (5) for modeling the production/growth trajectories. The least squares approach to fitting the model given by (3), (4), and (5) results in a **quadratic programming problem**. Though such estimates guarantee the monotonicity of the mean response curve, the estimates of $\mu_{i}\left(t\right)$ incurs small but non-negligible biases, particularly when the sample sizes are small. The monotonicity constraints on the mean trajectories and limited number of replicates both limit the application of classical large sample theory in dealing with the inference problem. Therefore, we develop a resampling-based strategy for statistical inference.

### 3.3. Key Parameters of Interest

We present the mathematical formulation of the inferential questions associated with the parameters of interest mentioned earlier.

1 **Optimal time-to-harvest: **$\theta_{i}=\min\left\{t:\mu_{i}\left(t\right)=c\right\}$ for i=1,⋯,I, where *c* is the prespecified cut-off level. The corresponding null hypothesis representing no factor effect on the “optimal time-to-harvest” is:
  (6) $$\begin{array}{c} \theta_{1}=\cdots =\theta_{I}. \end{array}$$
  With $\theta_{i}=\mu_{i}^{-1}\left(c\right)$ for some given cut-off level *c*, we are interested in testing the one-sided null hypotheses of the form $\theta_{1}\geq s_{1},\ldots ,\theta_{I}\geq s_{I}$ (here, the times $s_{1},\ldots ,s_{I}$ need not be equal). These hypotheses translate to the linear inequality constraints:
  (7) $$\begin{array}{c} \mu_{i}\left(s_{i}\right)\leq c\;\mathrm{for}\;\mathrm{all}\;i=1,\ldots ,I \end{array}$$
  Notice that, $\theta_{1}=\cdots =\theta_{I}$ is not a linear constraint.
  - However, the null hypothesis $\theta_{1}=\cdots =\theta_{I}=s_{0}$ can be translated to the equality constraints:
    (8) $$\begin{array}{c} \mu_{1}\left(s_{0}\right)=\cdots =\mu_{I}\left(s_{0}\right)=c. \end{array}$$
  - A composite null hypothesis of the form $s_{L}\leq \theta_{i}\leq s_{U}$ for all *i* can also be translated into linear inequality constraints
    (9) $$\begin{array}{c} \mu_{i}\left(s_{L}\right)\leq c\leq \mu_{i}\left(s_{U}\right) \end{array}$$
    for all *i*.
2 **Maximum production: **$\tau_{i}=\max_{t}\mu_{i}\left(t\right)$ for i=1,⋯,I. The regarding null hypotheses are
  (10) $$\begin{array}{c} \tau_{1}=\cdots =\tau_{I}. \end{array}$$
  or equivalently
  (11) $$\begin{array}{c} \mu_{1}\left(T_{\max}\right)=\mu_{2}\left(T_{\max}\right)=\cdots =\mu_{I}\left(T_{\max}\right). \end{array}$$
  where $T_{\max}$ is the largest time point during the experiment.
3 **Maximum “unweighted” productivity: **$\psi_{i}:=\max_{t}h_{i}^{u}\left(t\right)$, for i=1,…,I, where $h_{i}^{u}\left(t\right)=\frac{\mu_{i}\left(t\right)}{t+T_{i}^{\left(c\right)}}$ represents “unweighted” productivity of the *i*-th factor and $T_{i}^{\left(c\right)}$ is the number of days of cultivation before the induction. Here, “unweighted” means that we do not consider the rice cell fresh or dry weight. The corresponding null hypotheses are
  (12) $$\begin{array}{c} \psi_{1}=\cdots =\psi_{I} \end{array}$$
  or equivalently
  (13) $$\begin{array}{c} \max_{t}h_{1}\left(t\right)=\cdots =\max_{t}h_{I}\left(t\right). \end{array}$$
4 **Optimum stopping time:** Suppose the decision to harvest is taken based on the relative gradient of $\mu_{i}\left(t\right)$ (or, gradient of $\log\mu_{i}\left(t\right)$ ). Let $\gamma_{i}=\min\left\{t\geq T_{b}:\mu_{i}^{\prime }\left(t\right)/\mu_{i}\left(t\right)\leq \tilde{c}\right\}$ where $T_{b}$ is some constant baseline time and $\tilde{c}$ is a gradient threshold. We may be interested in testing hypotheses of the form $\gamma_{i}>s_{0}\;\mathrm{for}\;i=1,\ldots ,I$ where time $s_{0}$ is treated as the same for all *i* for simplicity. Since $\mu_{i}^{\prime }\left(t\right)/\mu_{i}\left(t\right)$ is not necessarily monotonic, this cannot be easily reduced to a simple set of inequality constraints. However, we may discretize time to a grid of the form $T_{b}=T_{1}<\cdots <T_{m}=s_{0}$ and then consider the slightly relaxed form of the hypothesis
  (14) $$\begin{array}{c} \mu_{i}^{\prime }\left(T_{j}\right)>\tilde{c}\mu_{i}\left(T_{j}\right)\;\;\mathrm{for}\;j=1,\ldots ,m,\;\mathrm{for}\;\mathrm{all}\;i. \end{array}$$
  In practice, *m* needs to be small for the feasibility of the optimization problem.

In a more general sense, if our interest is in testing for equality of $\Theta_{i}$ where $\Theta_{i}$ is a linear functional of $\mu_{i}\left(t\right)$, then we can design a procedure that imposes a less restrictive null hypothesis. This applies, for example, to the case when the maximum production level is the quantity of interest, since, under the monotonicity of $\mu_{i}$, this is simply the value at the maximum time point.

Specifically, suppose $\Theta_{i}=A\left(\mu_{i}\right)$ where *A* is a linear operator taking a scalar value, then $\Theta_{i}$ can be expressed as $\sum_{l}r_{l}\alpha_{il}$ where $r_{l}$ are some known coefficients and $\alpha_{il}$ represents the *l*-th spline coefficient of $\mu_{i}\left(t\right)=\sum_{l}\alpha_{il}B_{l}\left(t\right)$.

- Example 1: if $\Theta_{i}=\mu_{i}\left(T\right)$, then $r_{l}=B_{l}\left(T\right)$.
- Example 2: if $\Theta_{i}=\int \mu_{i}\left(t\right)dt$, then $r_{l}=\int B_{l}\left(t\right)dt$.

### 3.4. Statistical Inference Using Bootstrap

We mentioned earlier that the estimate ${\hat{\mu }}_{i}\left(t\right)$ obtained by imposing constraints (5) or (16) is not unbiased. What matters more, however, is that we have such a limited number of replicates that we cannot rely on *large sample theory* for making inferences. In view of this, it is imperative to adopt an inferential framework that does not depend too heavily either on the model assumptions, the methodology, or indeed the sample sizes.

Therefore, as a possible alternative, we propose to conduct hypothesis tests or construct confidence intervals for treatment-specific parameters. to be generically denoted by $\Theta_{i}$ (for *i*-th treatment), by making use of an appropriate resampling procedure. Below, we first describe the different types of resampling strategies that can be employed, depending on the data available. This is followed by specific choices for constructing confidence intervals or performing hypothesis tests involving one or many parameters.

#### 3.4.1. Resampling Strategies

Depending on the structure and amount of available data, we have several resampling or bootstrap strategies for computing the confidence intervals and performing hypothesis tests for the parameters of interest. The detailed procedures can be found in Section S1 in the Supplementary Materials.

**Nonparametric bootstrap with replicates:** This variant of resampling can be used when the number of replicates *n* is relatively but not extremely small, and thus we are able to construct the confidence interval for one parameter (e.g., the difference between a fixed pair of treatments). In some instances, specifically when the null hypotheses is formed by imposing linear equality constraints on the parameters, *bootstrap sampling distribution of the test statistic* (i.e., the histogram of the statistic used for testing the hypothesis, computed from the resampled data), under the null hypothesis can be calculated through a modified version of the nonparametric bootstrap procedure. This is done by replacing the original data with a set of “surrogate bootstrap data” that incorporates the constraints imposed by the null hypothesis. This enables an efficient computation of the *p*-values for the hypothesis being tested.

**Residual bootstrap with or without replicates:** This can be implemented whether there are replicates or not. The core idea here is to resample the residuals from fitting the model to the data. Note, however, that residual bootstrap is not effective particularly when the number of factors is small (say, if I=3, then the residual bootstrap method is not a good option since resampling does not capture the variability adequately as there are too few measurements to represent the true scale of variability of the data). Therefore, in practice, one needs to make a choice of bootstrap procedures based on the design of the experiment.

**Parametric bootstrap:** As an alternative to nonparametric or residual-based bootstrap, one can use the parametric bootstrap method if the model assumptions either can be validated (say, based on preliminary data) or if no significant departure from these is expected. As the variance of the noise is unknown, sample variance is typically used as a surrogate. When the number of replicates is small (e.g., n=1), and there are only a small number of treatments, neither nonparametric bootstrap nor residual bootstrap are feasible resampling strategies. In this challenging setting, a parametric bootstrap approach can still be used only if there is prior information on variability (see Section 2.2 for more details).

**Figure 7:**
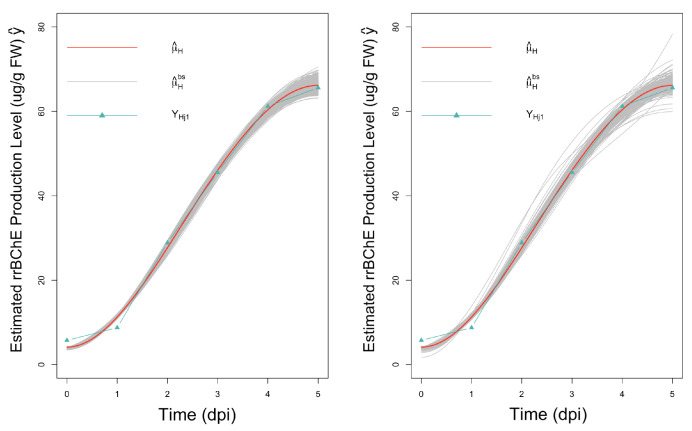
Observations, fitted curves and 500 bootstrap fitted curves for factor H; **left**: assuming normal noise; and **right**: assuming *t*-distributed noise.

In the proposed parametric bootstrap procedure, the observational noise is assumed to follow either a Gaussian distribution or a *t* distribution. For application to the rrBChE data, we use a scaled *t*-distribution with relatively low degrees of freedom, which allows for extreme values, thereby, reflecting the variability in the real data more effectively (Figure 7). By comparing the confidence intervals, we can see that the results in this real data application are similar regardless of the type of noise, thus, affirming a degree of robustness of the proposed method.

#### 3.4.2. Inference for a Single Parameter

**Construction of confidence intervals:** We propose two methods to construct the bootstrap confidence intervals:

1 *Percentile bootstrap confidence intervals*: We obtain percentile bootstrap confidence intervals for $\Theta_{i}$ and $\Theta_{i}-\Theta_{j}$ based on *B* bootstrap estimates of these parameters. The intervals are constructed by using appropriate quantiles of the bootstrap estimates ${\left\{{\hat{\Theta }}_{i}^{*b}\right\}}_{b=1}^{B}$ and ${\left\{{\hat{\Theta }}_{i}^{*b}-{\hat{\Theta }}_{j}^{*b}\right\}}_{b=1}^{B}$, respectively.
2 *Bias-corrected and accelerated bootstrap interval $BC_{a}$:* The percentile bootstrap confidence interval is only first-order accurate. Additionally, it does not correct for skewness of the sampling distribution. To address this, we use bias-corrected and accelerated bootstrap intervals [22], denoted by $BC_{a}$, which is not only second-order accurate but also corrects for the skewness in the sampling distribution.

**Computation of *p*-value associated with tests of hypothesis:** We propose two methods of obtaining approximate *p*-values using bootstrap. One is the percentile-*t* bootstrap, a general procedure, while the other one is a special case where the probability distribution of the test statistic under the null hypothesis can be calculated. For a general description of different types of bootstrap procedures and their theoretical validity, one may refer to [23].

*p-value computation by percentile-t bootstrap or percentile bootstrap:* The key idea behind computation of the *p*-values is to use the correspondence between hypothesis testing and confidence intervals. Specifically, the *p*-value is equal to $\eta^{*}$ where $\eta^{*}$ is the largest value of η such that the 100(1−η)% confidence interval contains the value of the parameter specified under the null hypothesis. We can make use of the percentile-*t* bootstrap procedure for constructing the confidence intervals for the parameters of interest for any given confidence level η and then “invert” these, as described above, to approximate the *p*-value. Alternatively, we may use the percentile bootstrap procedure (arguably less accurate) instead of percentile-*t* bootstrap, to compute the *p*-values, which incurs lower computational costs.

*p-value computation under the null distribution:* Instead of the indirect approach that relies on construction of bootstrap confidence intervals, in some instances, we can use a modified form of nonparametric bootstrap that enables generating samples under the distribution specified by the null hypothesis. Step 5 in Section S.2 indicates the method of computing *p*-values under this setup.

The key to successful application of this strategy is the ability to generate surrogate bootstrap data from the null distribution. This is feasible for example when the null hypotheses are specified by linear equality constraints on the parameters. It is reassuring that, for the real data analysis, where, due to the structure of the problem, we have two different ways of computing the *p*-values, viz., by inverting the bootstrap confidence intervals or by surrogate bootstrap data generated under the null hypothesis, the two versions of *p*-values are similar.

#### 3.4.3. Simultaneous Inference and Adjusting *p*-Values

When we perform simultaneous tests for multiple hypotheses of the form $H_{0}:\Theta_{i}=\Theta_{i^{\prime }}$ vs. $H_{1}:\Theta_{i}\neq \Theta_{i^{\prime }}$ for several pairs of treatments $1\leq i<i^{\prime }\leq I$, in order to control the *familywise type I error rate* (i.e., the probability of rejecting any of the null hypotheses incorrectly), it becomes necessary to adjust the level of significance of each individual test. This can be achieved by making use of the *Bonferroni procedure* or a *False Discovery Rate (FDR) control* procedure [24]. In the present setting, this requires computation of the *p*-values for each individual test using any one of the procedures described above, as appropriate.

Once we obtain the bootstrap *p*-values for the different tests, the *Benjamini–Hochberg (BH) procedure* for FDR control is used to first determine the significance level for each pairwise test for a given level of familywise significance. With this, we can adjust the confidence levels of the confidence intervals (*False Coverage-Statement Rate* (FCR)-Adjusted BH-Selected CIs) for the parameters accordingly [17]. We describe these procedures in Section S.3 of the Supplementary Material.

## 4. Conclusions

**Implications for scientific investigation:** The analysis presented here shows the merit of using advanced statistical techniques for answering critical questions about the comparative effectiveness of different experimental constructs in complex and expensive biological experiments where the data availability is limited. In particular, our study shows the capability of well-designed bootstrap methods for obtaining accurate confidence intervals for parameters of interest. In addition, it shows that the shapes of the mean protein production trajectories have a direct influence on the variability of the estimates of the “optimal time-to-harvest” and related parameters.

Moreover, the simulation study shows that the specialized resampling procedures provide a good description of this variability and therefore help experimenters to formulate an appropriate experimental design in terms of the number of time points and replicates for the experiment to achieve a desired level of accuracy. These assertions are further validated by an application of the methods to analyze the rrBChE data.

Furthermore, a salient feature of our framework is that the methodology works well even when the data are limited, and thus the standard large sample theory for statistical inference is not applicable, which is common in complicated biological experiments. In particular, the proposed methodology can help to suggest and validate the strategy of designing lengthy and expensive experiments where the number of replicates is limited. We also found that the two variants of bootstrap methods work well even when the amounts of data are limited. Specifically, if one is more confident in the model assumption, then parametric bootstrap is the preferred option. Otherwise, the residual bootstrap is a better choice as it yields more conservative confidence intervals.

In addition, our model can be used to obtain the optimal set of conditions to maximize parameters of interest, such as the optimum harvest time. When conditions are considered discrete, our resulting model framework has capabilities to provide statistical simultaneous inference to make comparisons. When the conditions are continuous, and the rate of change in the optimum harvest time is to be measured, we can modify the model by treating the optimum harvest time as a function of these conditions (treated as continuous covariates in the model).

Then, to optimize the optimum harvest time, we look to solve an equation setting the derivative (with respect to the conditions) of the optimum harvest time to zero. In that case, we can still apply an appropriately modified form of our bootstrap procedure to obtain confidence intervals for the parameters of interest. This can be also validated experimentally if enough resources are allowed in practice.

Finally, we discuss two possible directions in which the methodology presented here can be further enhanced to deal with additional questions about data generated from similar biological experiments.

**Extension 1:** The preceding analysis makes it clear that our framework is highly effective particularly when multiple process metrics are of interest. In addition to the parameters we discussed, our framework can be extended to other parameters, e.g., the average production and average “unweighted” productivity. The average production for the *i*-th factor is described as $\int_{T_{0}}^{T_{\max}}\mu_{i}\left(t\right)dt$ where $T_{0}$ is the starting point.

The corresponding null hypothesis is $\int_{T_{0}}^{T_{\max}}\mu_{1}\left(t\right)dt=\cdots =\int_{T_{0}}^{T_{\max}}\mu_{I}\left(t\right)dt$. Similarly, the average “unweighted” productivity is given by $\int_{T_{0}}^{T_{\max}}\mu_{1}\left(t\right)/\left(t+T_{i}^{\left(c\right)}\right)dt$ for the *i*-th factor. The null hypothesis is: $\int_{T_{0}}^{T_{\max}}\mu_{1}\left(t\right)/\left(t+T_{i}^{\left(c\right)}\right)dt=\cdots =\int_{T_{0}}^{T_{\max}}\mu_{I}\left(t\right)/\left(t+T_{i}^{\left(c\right)}\right)dt$. However, *p*-values cannot be calculated based on the null bootstrap distribution for average “unweighted” productivity because of nonlinearity. Instead, we may adopt the percentile bootstrap option to obtain *p*-values.

**Extension 2:** As we mentioned earlier, we used a simplified version of ANOVA framework. This can be extended to a two-factor ANOVA framework by incorporating the effect of the experimental condition, time and their interactions, with appropriate constraints. Such a modified model is able to leverage the impact of different factors and interactions of both factor and time and how different they are across factors. These are also key interests of experimental studies. In addition to Equations (3)–(5), we incorporate the factor effects and factor–time interaction by modeling $\alpha_{il}$ as follows:

(15) $$\begin{array}{c} \alpha_{il}=\beta +\eta_{i}+\xi_{l}+\delta_{il},\;i=1,\ldots ,I;l=1,\ldots ,L \end{array}$$

To ensure identifiability of the parameters, we impose the following linear constraints:

$$\begin{array}{cc} \sum_{i=1}^{I}\eta_{i} & =0,\;\sum_{l=1}^{L}\xi_{l}B_{l}\left(t^{*}\right)=0\\ \sum_{i=1}^{I}\delta_{il} & =0\;\mathrm{for}\;\mathrm{each}\;l,\;\sum_{l=1}^{L}\delta_{il}B_{l}\left(t^{*}\right)=0,\;\mathrm{for}\;\mathrm{each}\;i, \end{array}$$

where $t^{*}$ is an arbitrary but appropriately chosen point in [0,T]. Furthermore, the constraints (5) are equivalent to

(16) $$\xi_{l+1}-\xi_{l}+\delta_{i(l+1)}-\delta_{il}\geq 0,\;i=1,\ldots ,I;\;l=1,\ldots ,L-1.$$

The extended model given by (3), (4), (15) and (16) is a modified form of two-factor ANOVA and can be solved by quadratic programming. Statistical inference can be conducted by a natural extension of the resampling strategy proposed here.

## Figures and Tables

**Table 1:** The Percentile Bootstrap Confidence Interval (CI) and bias-corrected and accelerated bootstrap interval ($BC_{a}$) for c=10.3. The significance against $H_{0}:\theta_{i}=\theta_{j}$ is in red.

		CI Lower	CI Upper	CI Length	True	Mean	sd
Percentile Bootstrap CI	${\hat{\theta }}_{1}^{bs}$	5.820	7.027	1.207	6.378	6.320	0.327
	${\hat{\theta }}_{2}^{bs}$	5.892	9.000	3.108	7.387	7.739	0.995
	${\hat{\theta }}_{3}^{bs}$	4.730	5.162	0.432	4.910	4.917	0.113
	${\hat{\theta }}_{1}^{bs}-{\hat{\theta }}_{2}^{bs}$	−3.063	0.568	3.631	−1.009	−1.418	1.046
	${\hat{\theta }}_{2}^{bs}-{\hat{\theta }}_{3}^{bs}$	0.937	4.198	3.261	2.477	2.822	1.002
	${\hat{\theta }}_{3}^{bs}-{\hat{\theta }}_{1}^{bs}$	−2.171	−0.820	1.351	−1.468	−1.403	0.350
$BC_{a}$	${\hat{\theta }}_{1}^{bs}$	5.640	6.568	0.928			
	${\hat{\theta }}_{2}^{bs}$	6.036	9.000	2.964			
	${\hat{\theta }}_{3}^{bs}$	4.695	5.126	0.431			
	${\hat{\theta }}_{1}^{bs}-{\hat{\theta }}_{2}^{bs}$	−3.189	0.108	3.297			
	${\hat{\theta }}_{2}^{bs}-{\hat{\theta }}_{3}^{bs}$	1.117	4.252	3.135			
	${\hat{\theta }}_{3}^{bs}-{\hat{\theta }}_{1}^{bs}$	−1.766	−0.525	1.241			

**Table 2:** Using residual(nonparametric) bootstrap methods: False coverage-statement rate (FCR)—Adjusted BH-Selected CIs for selected parameters indicated by the percentile bootstrap CI; All confidence intervals above show significance against $H_{0}:\tau_{i}=\tau_{j}$.

	*p*-Value by Percentile Bootstrap CI	CI Lower	CI Upper	CI Length	Mean	sd	Est
${\hat{\tau }}_{A}^{bs}-{\hat{\tau }}_{H}^{bs}$	<$10^{-5}$	−28.059	−10.204	17.856	−19.298	3.624	−19.186
${\hat{\tau }}_{B}^{bs}-{\hat{\tau }}_{H}^{bs}$	<$10^{-5}$	−25.265	−8.512	16.753	−17.707	3.213	−18.115
${\hat{\tau }}_{C}^{bs}-{\hat{\tau }}_{H}^{bs}$	<$10^{-5}$	−26.081	−9.485	16.596	−18.197	3.379	−18.347
${\hat{\tau }}_{D}^{bs}-{\hat{\tau }}_{F}^{bs}$	0.008	0.659	17.297	16.638	8.125	3.348	7.544
${\hat{\tau }}_{D}^{bs}-{\hat{\tau }}_{H}^{bs}$	<$10^{-5}$	−19.830	−3.745	16.086	−12.414	3.191	−12.679
${\hat{\tau }}_{E}^{bs}-{\hat{\tau }}_{H}^{bs}$	<$10^{-5}$	−24.668	−7.003	17.665	−15.496	3.489	−15.233
${\hat{\tau }}_{F}^{bs}-{\hat{\tau }}_{H}^{bs}$	<$10^{-5}$	−29.730	−11.710	18.020	−20.539	3.677	−20.223
${\hat{\tau }}_{G}^{bs}-{\hat{\tau }}_{H}^{bs}$	<$10^{-5}$	−24.095	−7.635	16.460	−16.482	3.187	−16.687

**Table 3:** Using parametric bootstrap: False coverage-statement rate (FCR)—Adjusted BH-Selected CIs for selected parameters indicated by the percentile bootstrap CI; All confidence intervals above show significance against $H_{0}:\tau_{i}=\tau_{j}$.

	*p*-Value by Percentile Bootstrap CI	CI Lower	CI Upper	CI Length	Mean	sd	Est
${\hat{\tau }}_{A}^{bs}-{\hat{\tau }}_{D}^{bs}$	0.002	−10.778	−3.105	7.674	−6.578	1.545	−6.507
${\hat{\tau }}_{A}^{bs}-{\hat{\tau }}_{H}^{bs}$	<$10^{-5}$	−23.481	−15.508	7.974	−19.277	1.731	−19.186
${\hat{\tau }}_{B}^{bs}-{\hat{\tau }}_{D}^{bs}$	0.006	−9.552	−1.525	8.026	−5.301	1.525	−5.436
${\hat{\tau }}_{B}^{bs}-{\hat{\tau }}_{H}^{bs}$	<$10^{-5}$	−22.067	−14.185	7.882	−17.999	1.653	−18.115
${\hat{\tau }}_{C}^{bs}-{\hat{\tau }}_{D}^{bs}$	0.006	−10.075	−1.356	8.719	−5.662	1.644	−5.668
${\hat{\tau }}_{C}^{bs}-{\hat{\tau }}_{H}^{bs}$	<$10^{-5}$	−22.761	−14.358	8.403	−18.361	1.709	−18.347
${\hat{\tau }}_{D}^{bs}-{\hat{\tau }}_{F}^{bs}$	0.002	2.768	12.825	10.057	7.680	1.828	7.544
${\hat{\tau }}_{D}^{bs}-{\hat{\tau }}_{G}^{bs}$	0.022	0.383	8.532	8.149	4.106	1.553	4.008
${\hat{\tau }}_{D}^{bs}-{\hat{\tau }}_{H}^{bs}$	0.002	−16.687	−8.478	8.209	−12.698	1.765	−12.679
${\hat{\tau }}_{E}^{bs}-{\hat{\tau }}_{F}^{bs}$	0.018	0.121	10.278	10.157	4.859	1.877	4.990
${\hat{\tau }}_{E}^{bs}-{\hat{\tau }}_{H}^{bs}$	<$10^{-5}$	−20.403	−11.297	9.106	−15.519	1.925	−15.233
${\hat{\tau }}_{F}^{bs}-{\hat{\tau }}_{H}^{bs}$	<$10^{-5}$	−25.471	−15.836	9.635	−20.378	1.872	−20.223
${\hat{\tau }}_{G}^{bs}-{\hat{\tau }}_{H}^{bs}$	<$10^{-5}$	−20.893	−12.667	8.225	−16.804	1.648	−16.687

**Table 4:** Conditions used in bioreactor runs A–H where the agitation rate and temperature were maintained at 75 rpm and 27 °C, respectively, in all runs. The aeration rate was maintained at 0.2 vvm (volume of sparged gas per working volume per minute) in runs A–F and 0.2–0.4 vvm in runs G and H (reproduced from [11]).

Experiment	%DO during Growth Phase	%DO during Induction Phase	Media Exchange
A	40	10	Yes
B	40	20	Yes
C	40	30	Yes
D	40	40	Yes
E	40	Uncontrolled	Yes
F	40	40	No
G	Uncontrolled	Uncontrolled	No
H ${}^{1}$	Uncontrolled	Uncontrolled	No

^1^ Initial sucrose concentration in the medium in run H was reduced to 15 g/L instead of 30 g/L used in other runs. DO, dissolved oxygen.

## Data Availability

The experimental data has been open to public and can be found through https://orcid.org/0000-0001-6750-4375 (accessed on 11 April 2022).

## Supplementary Materials

The following are available at https://www.mdpi.com/article/10.3390/ijms23147628/s1.
