# Supplementary material for: Analysis of Variability of Functionals of Recombinant Protein Production Trajectories Based on Limited Data
Source: Int J Mol Sci. 2022 Jul 10;23(14):7628. doi: 10.3390/ijms23147628 (PMC9317391; doi:10.3390/ijms23147628)
Supplement: Supplementary file 1 [file ijms-23-07628-s001.zip › ijms-1701122-supplementary.pdf]

Supplementary Material: "Analysis of variability of functionals of recombinant protein production trajectories based on limited data"

Shuting Liao*[1], Kantharakorn Macharoen*[2,5],
Karen A. McDonald[2,3], Somen Nandi†[2,3], and Debashis Paul†[4]

[1] *Graduate Group in Biostatistics, University of California, Davis, CA 95616, USA*
[2] *Department of Chemical Engineering, University of California, Davis, CA 95616, USA*
[3] *Global HealthShare, University of California, Davis, CA 95616, USA*
[4] *Department of Statistics, University of California, Davis, CA 95616, USA*
[5] Current affiliation: *Department of Chemical Engineering, King Mongkut's University of Technology Thonburi, Thailand*

Supplementary Materials

S.1 Details on resampling strategies

Nonparametric bootstrap with replicates When the number of replicates n is relatively but not extremely small, we construct the confidence interval for one parameter (e.g., the difference between a fixed pair of treatments) as follows, using a nonparametric bootstrap procedure. We re-sample with replacement randomly, samples of size n, from the collection of observations $\{Y_{ijk}\}_{k=1}^{n}$ for each treatment-time pair (i, j). We repeat this process B times and denote $\{Y_{ijk}^{*b}\}_{k=1}^{n}$ as the b-th sample corresponding to pair (i, j). Then we fit the model given by equations (1), (2), (4) and (5) and obtain a set of estimated parameters we are interested, denoted by $\hat{\Theta}_1^{*b}, \ldots, \hat{\Theta}_I^{*b}$.

Parametric bootstrap with replicates (without prior information on variability) The procedures for parametric bootstrap method are as follows:

- Calculate the sample variance for the i-th treatment at time t_j based on n_i replicates (for balanced data: $n_i = n$): $\hat{s}_{ij}^2 = \frac{1}{n_i-1}\sum_{k=1}^{n_i}(Y_{ijk} - \hat{\mu}_i(t_j))^2$
- Compute the pooled variance $\hat{s}_j^2 = \frac{\sum_i^I (n_i-1)\hat{s}_{ij}^2}{\sum_i^I (n_i-1)}$

*Joint first author.

†Corresponding authors: snandi@ucdavis.edu, debpaul@ucdavis.edu

- Simulate parametric bootstrap samples $Y_{ijk}^{*pb} = \hat{\mu}_i(t_j) + \epsilon_{ijk}^{*pb}$ where $\epsilon_{ijk}^{*pb} \sim N(0, \hat{s}_j^2)$.

Parametric bootstrap without replicates (with prior information on variability) Note that here we make use of pooled variance $\hat{SD^2}_{.j}$ for all treatments at time j based on $\{SD_{ij}^2\}_{i,j}$. That is, $\hat{SD^2}_{.j} = \frac{1}{I}\sum_i SD_{ij}^2$. We use two different strategies to generate bootstrap samples. One is using normal distribution while the other one is t-distribution, which allows for more extreme values.

- **Gaussian noise:** At each time $b = 1, \ldots, B$, we generate samples by $\mu_{ij}^{*b} = \hat{\mu}_{ij} + \epsilon_{ij}^{*b}$, where $\epsilon_{ij}^{*b} \sim N(0, \hat{SD^2}_{.j})$. We use $\{\mu_{ij}^{*b}\}_{ij}$ for model fitting, leading to $\{\hat{\Theta}_i^{*b}\}_{b=1}^B$.

- ***t*-distributed noise:** Instead of assuming noise approximately follows normal distribution, we use t-distribution to allow more extreme values. We obtain the scale parameter by $\hat{SD^2}_{.j} = \sigma_j^2 \times \frac{df}{df-2}$ where df is the degree of freedom of t-distribution; $\hat{SD^2}_{.j}$ is the measurement error (pooled variance). Then we generate noise which follows $\sigma_j t_{df}$. Here we assume $df = 3$, which means the variance is finite.

Residual bootstrap with or without replicates The general route of generating residual bootstrap samples with or without replicates (i.e., $n \geq 1$)is as follows:

1. Suppose $\{Y_{ijk}\}$ as the raw data and obtain the $\hat{\mu}_i(t_j)$;
2. Obtain $\hat{e}_{ijk} = Y_{ijk} - \hat{\mu}_i(t_j)$, which is biased due to the framework;
3. For each j, obtain e_{ijk}^{*b} resampled from $\{\hat{e}_{ijk} - \bar{e}_{.j.}\}$ for $i = 1, \ldots, I$ and $k = 1, \ldots, n$ where $\bar{e}_{.j.} = \frac{1}{I \times n}\sum_i \sum_k \hat{e}_{ijk}$. Note that $\{\hat{e}_{ijk} - \bar{e}_{.j.}\}$ will sum to 0 for each j.
4. Generate residual bootstrap samples: $Y_{ijk}^{*b} = \hat{\mu}_i(t_j) + e_{ijk}^{*b}$.

Nonparametric bootstrap based on the null distribution:

In each of the aforementioned hypothesis testing problems, the estimation problem under the null hypothesis remains a quadratic programming problem, with linear equality or inequality constraints on the parameters. In each case, the key parameters involved can be expressed in the form

$$\sum_{l=1}^{L} r_l \alpha_{il} \tag{S.1}$$

where r_l's are some constants and α_{il} is the coefficient of $\mu_i(t)$ associated with the l-th B-spline $B_l(t)$. Since the estimation problem is a quadratic programming problem, we can use the fitted trajectories to construct surrogate bootstrap data and then make use of this to compute the null distribution of the test statistics, and thereby obtain the p-value of the test. The surrogate data are computed as

$$\tilde{Y}_{ijk} = e^b_{ijk} + \tilde{\mu}_i(t_j) \tag{S.2}$$

where e^b_{ijk} are sampled by bootstrap procedures based on observational residuals $\{Y_{ijk} - \hat{\mu}_i(t_j)\}$, $\tilde{\mu}_i$ is the estimate of μ_i under the constraints imposed by the null hypothesis. The detailed algorithm using the null distribution is shown at the end of Section S.2.

S.2 Computation of p-value associated with tests of hypothesis

p-value computation by percentile-t bootstrap: Suppose we are performing pairwise tests for equality of the Θ_i's, where Θ_i is some functional of μ_i , by doing percentile-t bootstrap. The $100(1-\alpha)\%$ bootstrap confidence intervals for $\delta_{ij} = \Theta_i - \Theta_j$ are of the form

$$[\hat{\delta}_{ij} - q_{1-\alpha/2}\hat{\sigma}_{ij}, \hat{\delta}_{ij} - q_{\alpha/2}\hat{\sigma}_{ij}] \tag{S.3}$$

where $\delta_{ij} = \Theta_i - \Theta_j$, $\hat{\sigma}_{ij}$ is the estimated standard error of $\hat{\delta}_{ij}$ and q_p is the p-th quantile of the bootstrap distribution of the t-statistics

$$(\hat{\delta}^{*b}_{ij} - \hat{\delta}_{ij})/\hat{\sigma}^{*b}_{ij} \tag{S.4}$$

where $\hat{\delta}^{*b}_{ij}$ is the bootstrap estimate of δ_{ij} and $\hat{\sigma}^{*b}_{ij}$ is the bootstrap estimate of its standard error. Then, we may define a bootstrap p-value for testing $H^{ij}_0 : \Theta_i = \Theta_j$ to be the largest value of α such that the aforementioned $100(1-\alpha)\%$ bootstrap confidence interval contains 0. Note that $\hat{\sigma}_{ij}$ and $\hat{\sigma}^{*b}_{ij}$ can be estimated by using "double bootstrap" procedure described below (assuming only one replicate):

1. Generate bootstrap samples $\{Y^{*b}_{ij}\}^B_{b=1}$, where B is large (say $B = 1000$) and Y^{*b}_{ij} is the b-th bootstrap measurement on i-th treatment at time t_j.

2. Let $\hat{\Theta}^{*b}_i$ be the corresponding bootstrap estimate of Θ_i, and correspondingly, $\hat{\delta}^{*b}_{ik} = \hat{\Theta}^{*b}_i - \hat{\Theta}^{*b}_k$.

3. Estimate σ^2_{ik} as follows:
$$\hat{\sigma}^2_{ik} = (B-1)^{-1}\sum_{b=1}^{B}(\hat{\delta}^{*b}_{ik} - \bar{\delta}^*_{ik})^2$$
where $\bar{\delta}^*_{ik} = B^{-1}\sum_{b=1}^{B}\hat{\delta}^{*b}_{ik}$ is a bootstrap estimate of $E(\hat{\delta}_{ik})$.

4. In the percentile-t bootstrap procedure, we compute a bootstrap estimate of $\hat{\sigma}_{ik}$, namely, $\hat{\sigma}^{*b}_{ik}$, corresponding to the b-th bootstrap sample used for inference. This will involve repeating the procedure as described above, but now treating the data for the b-th bootstrap sample (Y^{*b}_{ij}) as the raw data.

5. The procedure for computing $\hat{\sigma}_{ik}^{*b}$ is actually a so-called "double bootstrap" procedure. Again, these bootstrap samples for each b will have to be generated independently. So, this is clearly a computationally intensive procedure.

p-value computation by percentile bootstrap The percentile bootstrap is less computationally intensive since it does not require computation of $\hat{\sigma}_{ik}^{*b}$ for each bootstrap sample, and so there is no need for the second layer of bootstrap. We only need to find the bootstrap p-value to be the largest value of α such that the $100(1-\alpha)\%$ bootstrap confidence interval contains 0.

p-value computation by nonparametric bootstrap based on the null distribution Here are the detailed steps of the bootstrap procedure for approximating the sampling distribution under the null hypothesis of no difference across the different treatments:

1. Use the current method to compute $\hat{\mu}_i(t)$ for the different treatments.

2. Obtain the fitted curves $\tilde{\mu}_i(t)$ for the different treatments from the original data by imposing the additional constraint on the coefficients (α_{il}) that

$$\Theta_1 = \cdots = \Theta_I, \text{ that is }, \sum_l r_l \alpha_{1l} = \cdots = \sum_l r_l \alpha_{Il}$$

3. Create surrogate bootstrap data $\tilde{Y}_{ijk}^b = e_{ijk}^b + \tilde{\mu}_i(t_j), i = 1, \ldots, I, j = 1, \ldots, J$, where e_{ijk}^b are generated based on parametric (t-distribution) or non-parametric (sampled from $= Y_{ijk} - \hat{\mu}_i(t_j)$) bootstrap sampling distribution. $\{\{\tilde{Y}_{ijk}\}^b\}$ are the bootstrap samples under the null hypothesis.

4. Obtain bootstrap estimates $\{\hat{\mu}_i^{*b}(t)\}_{b=1}^B$. This leads us to a set of bootstrap estimates of Θ_i, $\{\Theta_i^{*b}\}_{b=1}^B$.

5. Use the bootstrap sampling distribution computed in Step 4 to obtain the p-values for testing $\Theta_i = \Theta_{i'}$ for all pairs (i, i').

Notice that Step 2 of the above procedure imposes linear equality constraints on the parameters (α_{il}), along with the original monotonicity and non-negativity constraints, and therefore the resulting least squares problem can still be solved by quadratic programming. Thus, Step 2 obtains least squares estimates of $\mu_1, \cdots, \mu_I$, under the monotonicity constraint and the additional requirement (null hypothesis) that $\Theta_1 = \cdots = \Theta_I$. This is clearly less stringent than requiring that μ_i's are all equal. In step 5, for example, if $\tau_i = \max_t \mu_i(t)$, then the p-value for the test will be computed as the fraction of times $|\hat{\tau}_i^* - \hat{\tau}_k^*| > |\hat{\tau}_i - \hat{\tau}_k|$. Here, $\hat{\tau}_i = \max_t \hat{\mu}_i(t)$ (with $\hat{\mu}_i$ computed in Step 1) and $\hat{\tau}_i^{*b} = \max_t \hat{\mu}_i^{*b}(t)$ (with $\hat{\mu}_i^{*b}$ computed in Step 4).

S.3 Simultaneous inference

1. Sort the p-values used for testing the m hypotheses regarding the parameters, $p_{(1)} \leq \cdots \leq p_{(M)}$, where M is the number of tests.

2. Calculate $R = \max\{i : p_{(i)} \leq \frac{i}{M}\alpha\}$ where α is the significance level.

3. Select R parameters for which $p_{(i)} \leq R\frac{\alpha}{M}$, corresponding to the rejected hypothesis.

4. Construct a $1 - R\frac{\alpha}{m}$ CI for each parameter selected.

S.4 Figures

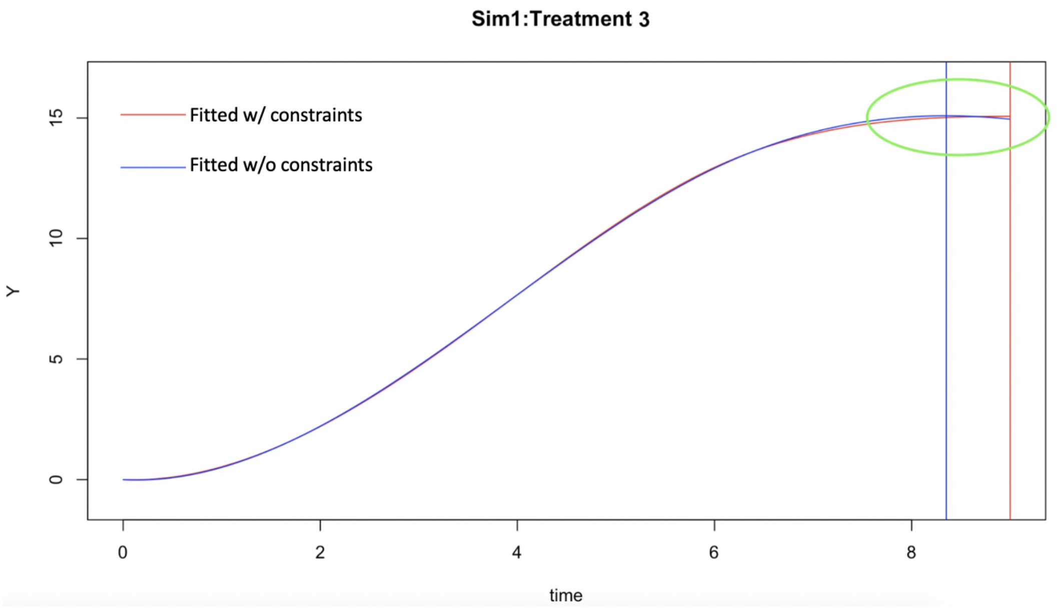

Figure S1: In the simulation study of Treatment 3: blue curve represents unconstrained estimation while the red curve is constrained estimation. Decreasing pattern shown at the end of time point in treatment 3; the peak value of unconstrained estimation is shown by blue vertical line while the red vertical line for the peak of constrained one.

S.5 Tables

	p-value based on the null bootstrap distribution	CI lower	CI upper	CI length	mean	sd	est
$\hat{\tau}_A^{bs} - \hat{\tau}_H^{bs}$	$< 10^{-5}$	-28.082	-9.975	18.107	-19.298	3.624	-19.186
$\hat{\tau}_B^{bs} - \hat{\tau}_H^{bs}$	$< 10^{-5}$	-25.337	-8.495	16.842	-17.707	3.213	-18.115
$\hat{\tau}_C^{bs} - \hat{\tau}_H^{bs}$	$< 10^{-5}$	-26.293	-9.439	16.853	-18.197	3.379	-18.347
$\hat{\tau}_D^{bs} - \hat{\tau}_H^{bs}$	$< 10^{-5}$	-20.150	-3.602	16.547	-12.414	3.191	-12.679
$\hat{\tau}_E^{bs} - \hat{\tau}_H^{bs}$	$< 10^{-5}$	-24.672	-6.965	17.708	-15.496	3.489	-15.233
$\hat{\tau}_F^{bs} - \hat{\tau}_H^{bs}$	$< 10^{-5}$	-29.974	-11.644	18.330	-20.539	3.677	-20.223
$\hat{\tau}_G^{bs} - \hat{\tau}_H^{bs}$	$< 10^{-5}$	-24.125	-7.616	16.509	-16.482	3.187	-16.687

Table S1: Using **residual** (nonparametric) bootstrap methods: False coverage-statement rate (FCR) - Adjusted BH-Selected CIs for selected parameters indicated by **the null bootstrap distribution**; All confidence intervals above show significance against $H_0 : \tau_i = \tau_j$

	p-value based on the null bootstrap distribution	CI lower	CI upper	CI length	mean	sd	est
$\hat{\tau}_A^{bs} - \hat{\tau}_D^{bs}$	0.003	-10.778	-3.105	7.674	-6.578	1.545	-6.507
$\hat{\tau}_A^{bs} - \hat{\tau}_H^{bs}$	$< 10^{-5}$	-23.481	-15.508	7.974	-19.277	1.731	-19.186
$\hat{\tau}_B^{bs} - \hat{\tau}_D^{bs}$	0.008	-9.552	-1.525	8.026	-5.301	1.525	-5.436
$\hat{\tau}_B^{bs} - \hat{\tau}_H^{bs}$	$< 10^{-5}$	-22.067	-14.185	7.882	-17.999	1.653	-18.115
$\hat{\tau}_C^{bs} - \hat{\tau}_D^{bs}$	0.006	-10.075	-1.356	8.719	-5.662	1.644	-5.668
$\hat{\tau}_C^{bs} - \hat{\tau}_H^{bs}$	$< 10^{-5}$	-22.761	-14.358	8.403	-18.361	1.709	-18.347
$\hat{\tau}_D^{bs} - \hat{\tau}_F^{bs}$	0.003	2.768	12.825	10.057	7.680	1.828	7.544
$\hat{\tau}_D^{bs} - \hat{\tau}_G^{bs}$	0.019	0.383	8.532	8.149	4.106	1.553	4.008
$\hat{\tau}_D^{bs} - \hat{\tau}_H^{bs}$	$< 10^{-5}$	-16.687	-8.478	8.209	-12.698	1.765	-12.679
$\hat{\tau}_E^{bs} - \hat{\tau}_F^{bs}$	0.022	0.121	10.278	10.157	4.859	1.877	4.990
$\hat{\tau}_E^{bs} - \hat{\tau}_H^{bs}$	$< 10^{-5}$	-20.403	-11.297	9.106	-15.519	1.925	-15.233
$\hat{\tau}_F^{bs} - \hat{\tau}_H^{bs}$	$< 10^{-5}$	-25.471	-15.836	9.635	-20.378	1.872	-20.223
$\hat{\tau}_G^{bs} - \hat{\tau}_H^{bs}$	$< 10^{-5}$	-20.893	-12.667	8.225	-16.804	1.648	-16.687

Table S2: Using **parametric** bootstrap: False coverage-statement rate (FCR) - Adjusted BH-Selected CIs for selected parameters indicated by **the null bootstrap distribution**; All confidence intervals above show significance against $H_0 : \tau_i = \tau_j$

	p-value by percentile bootstrap CI	CI lower	CI upper	CI length	mean	sd	est
$\hat{\psi}_A^{bs} - \hat{\psi}_F^{bs}$	$< 10^{-5}$	0.203	1.510	1.307	0.822	0.271	0.841
$\hat{\psi}_A^{bs} - \hat{\psi}_G^{bs}$	$< 10^{-5}$	1.163	2.082	0.919	1.698	0.204	1.714
$\hat{\psi}_A^{bs} - \hat{\psi}_H^{bs}$	$< 10^{-5}$	-1.385	-0.181	1.204	-0.796	0.260	-0.797
$\hat{\psi}_B^{bs} - \hat{\psi}_F^{bs}$	$< 10^{-5}$	0.717	1.874	1.157	1.180	0.256	1.252
$\hat{\psi}_B^{bs} - \hat{\psi}_G^{bs}$	$< 10^{-5}$	1.650	2.478	0.827	2.056	0.189	2.124
$\hat{\psi}_C^{bs} - \hat{\psi}_F^{bs}$	$< 10^{-5}$	0.571	1.775	1.204	1.029	0.259	1.086
$\hat{\psi}_C^{bs} - \hat{\psi}_G^{bs}$	$< 10^{-5}$	1.501	2.349	0.848	1.905	0.193	1.959
$\hat{\psi}_D^{bs} - \hat{\psi}_F^{bs}$	$< 10^{-5}$	0.732	1.863	1.131	1.161	0.248	1.219
$\hat{\psi}_D^{bs} - \hat{\psi}_G^{bs}$	$< 10^{-5}$	1.653	2.438	0.786	2.036	0.177	2.092
$\hat{\psi}_E^{bs} - \hat{\psi}_F^{bs}$	$< 10^{-5}$	0.586	1.852	1.266	1.162	0.262	1.096
$\hat{\psi}_E^{bs} - \hat{\psi}_G^{bs}$	$< 10^{-5}$	1.496	2.409	0.914	2.038	0.203	1.968
$\hat{\psi}_F^{bs} - \hat{\psi}_G^{bs}$	$< 10^{-5}$	0.267	1.209	0.942	0.876	0.210	0.872
$\hat{\psi}_F^{bs} - \hat{\psi}_H^{bs}$	$< 10^{-5}$	-2.274	-1.019	1.254	-1.619	0.267	-1.638
$\hat{\psi}_G^{bs} - \hat{\psi}_H^{bs}$	$< 10^{-5}$	-2.875	-1.977	0.898	-2.495	0.201	-2.511

Table S3: Using **residual** bootstrap method: False coverage-statement rate (FCR) -Adjusted BH-Selected CIs for selected parameters indicated by **the percentile bootstrap CIs**; All confidence intervals above show significance against $H_0: \psi_i = \psi_j$

	p-value based on the null bootstrap distribution	CI lower	CI upper	CI length	mean	sd	est
$\hat{\psi}_A^{bs} - \hat{\psi}_B^{bs}$	0.004	-0.644	-0.174	0.471	-0.401	0.110	-0.410
$\hat{\psi}_A^{bs} - \hat{\psi}_D^{bs}$	0.008	-0.594	-0.158	0.436	-0.369	0.106	-0.378
$\hat{\psi}_A^{bs} - \hat{\psi}_E^{bs}$	0.030	-0.611	-0.034	0.578	-0.282	0.137	-0.255
$\hat{\psi}_A^{bs} - \hat{\psi}_F^{bs}$	$< 10^{-5}$	0.507	1.127	0.620	0.823	0.135	0.841
$\hat{\psi}_A^{bs} - \hat{\psi}_G^{bs}$	$< 10^{-5}$	1.501	1.894	0.393	1.698	0.095	1.714
$\hat{\psi}_A^{bs} - \hat{\psi}_H^{bs}$	0.002	-1.055	-0.568	0.486	-0.809	0.123	-0.797
$\hat{\psi}_B^{bs} - \hat{\psi}_F^{bs}$	$< 10^{-5}$	0.940	1.488	0.549	1.224	0.123	1.252
$\hat{\psi}_B^{bs} - \hat{\psi}_G^{bs}$	$< 10^{-5}$	1.914	2.261	0.347	2.099	0.081	2.124
$\hat{\psi}_B^{bs} - \hat{\psi}_H^{bs}$	0.006	-0.628	-0.205	0.423	-0.407	0.112	-0.387
$\hat{\psi}_C^{bs} - \hat{\psi}_F^{bs}$	$< 10^{-5}$	0.763	1.312	0.549	1.055	0.123	1.086
$\hat{\psi}_C^{bs} - \hat{\psi}_G^{bs}$	$< 10^{-5}$	1.717	2.096	0.379	1.929	0.084	1.959
$\hat{\psi}_C^{bs} - \hat{\psi}_H^{bs}$	0.002	-0.813	-0.374	0.439	-0.577	0.112	-0.552
$\hat{\psi}_D^{bs} - \hat{\psi}_F^{bs}$	$< 10^{-5}$	0.885	1.448	0.563	1.192	0.122	1.219
$\hat{\psi}_D^{bs} - \hat{\psi}_G^{bs}$	$< 10^{-5}$	1.885	2.212	0.327	2.067	0.078	2.092
$\hat{\psi}_D^{bs} - \hat{\psi}_H^{bs}$	0.002	-0.672	-0.246	0.426	-0.439	0.112	-0.419
$\hat{\psi}_E^{bs} - \hat{\psi}_F^{bs}$	$< 10^{-5}$	0.800	1.426	0.626	1.105	0.143	1.096
$\hat{\psi}_E^{bs} - \hat{\psi}_G^{bs}$	$< 10^{-5}$	1.784	2.241	0.458	1.979	0.110	1.968
$\hat{\psi}_E^{bs} - \hat{\psi}_H^{bs}$	0.008	-0.778	-0.236	0.542	-0.527	0.138	-0.542
$\hat{\psi}_F^{bs} - \hat{\psi}_G^{bs}$	$< 10^{-5}$	0.629	1.145	0.516	0.875	0.112	0.872
$\hat{\psi}_F^{bs} - \hat{\psi}_H^{bs}$	$< 10^{-5}$	-1.906	-1.342	0.564	-1.631	0.135	-1.638
$\hat{\psi}_G^{bs} - \hat{\psi}_H^{bs}$	$< 10^{-5}$	-2.696	-2.323	0.373	-2.506	0.097	-2.511

Table S4: Using **parametric** bootstrap method: False coverage-statement rate (FCR) - Adjusted BH-Selected CIs for selected parameters indicated by **the percentile bootstrap CIs**; All confidence intervals above show significance against $H_0 : \psi_i = \psi_j$

	CI lower	CI upper	CI length	mean	sd	est
$\hat{\theta}^{bs}_A$	3.493	3.849	0.355	3.642	0.096	3.619
$\hat{\theta}^{bs}_B$	2.758	2.978	0.220	2.841	0.064	2.813
$\hat{\theta}^{bs}_C$	2.958	3.238	0.280	3.081	0.077	3.063
$\hat{\theta}^{bs}_D$	2.513	2.678	0.165	2.587	0.046	2.588
$\hat{\theta}^{bs}_E$	3.138	3.463	0.325	3.278	0.081	3.268
$\hat{\theta}^{bs}_F$	4.174	4.484	0.310	4.318	0.077	4.314
$\hat{\theta}^{bs}_G$	2.673	2.878	0.205	2.763	0.055	2.748
$\hat{\theta}^{bs}_H$	2.578	2.743	0.165	2.659	0.042	2.658
$\hat{\theta}^{bs}_A - \hat{\theta}^{bs}_B$	0.591	1.021	0.430	0.801	0.116	0.806
$\hat{\theta}^{bs}_A - \hat{\theta}^{bs}_C$	0.315	0.801	0.485	0.561	0.122	0.556
$\hat{\theta}^{bs}_A - \hat{\theta}^{bs}_D$	0.876	1.271	0.395	1.055	0.107	1.031
$\hat{\theta}^{bs}_A - \hat{\theta}^{bs}_E$	0.130	0.631	0.501	0.364	0.125	0.350
$\hat{\theta}^{bs}_A - \hat{\theta}^{bs}_F$	-0.911	-0.440	0.470	-0.676	0.122	-0.696
$\hat{\theta}^{bs}_A - \hat{\theta}^{bs}_G$	0.686	1.086	0.400	0.879	0.110	0.871
$\hat{\theta}^{bs}_A - \hat{\theta}^{bs}_H$	0.806	1.216	0.410	0.983	0.105	0.961
$\hat{\theta}^{bs}_B - \hat{\theta}^{bs}_C$	-0.430	-0.060	0.370	-0.240	0.102	-0.250
$\hat{\theta}^{bs}_B - \hat{\theta}^{bs}_D$	0.130	0.415	0.285	0.254	0.079	0.225
$\hat{\theta}^{bs}_B - \hat{\theta}^{bs}_E$	-0.631	-0.245	0.385	-0.437	0.103	-0.455
$\hat{\theta}^{bs}_B - \hat{\theta}^{bs}_F$	-1.662	-1.281	0.380	-1.477	0.102	-1.502
$\hat{\theta}^{bs}_B - \hat{\theta}^{bs}_G$	-0.075	0.240	0.315	0.078	0.087	0.065
$\hat{\theta}^{bs}_B - \hat{\theta}^{bs}_H$	0.060	0.335	0.275	0.182	0.078	0.155
$\hat{\theta}^{bs}_C - \hat{\theta}^{bs}_D$	0.335	0.691	0.355	0.494	0.089	0.475
$\hat{\theta}^{bs}_C - \hat{\theta}^{bs}_E$	-0.430	0.025	0.455	-0.197	0.113	-0.205
$\hat{\theta}^{bs}_C - \hat{\theta}^{bs}_F$	-1.441	-1.006	0.435	-1.237	0.109	-1.251
$\hat{\theta}^{bs}_C - \hat{\theta}^{bs}_G$	0.145	0.506	0.360	0.318	0.094	0.315
$\hat{\theta}^{bs}_C - \hat{\theta}^{bs}_H$	0.260	0.621	0.360	0.422	0.089	0.405
$\hat{\theta}^{bs}_D - \hat{\theta}^{bs}_E$	-0.891	-0.521	0.370	-0.691	0.093	-0.681
$\hat{\theta}^{bs}_D - \hat{\theta}^{bs}_F$	-1.907	-1.552	0.355	-1.731	0.089	-1.727
$\hat{\theta}^{bs}_D - \hat{\theta}^{bs}_G$	-0.315	-0.050	0.265	-0.176	0.072	-0.160
$\hat{\theta}^{bs}_D - \hat{\theta}^{bs}_H$	-0.190	0.045	0.235	-0.072	0.063	-0.070
$\hat{\theta}^{bs}_E - \hat{\theta}^{bs}_F$	-1.246	-0.821	0.425	-1.040	0.111	-1.046
$\hat{\theta}^{bs}_E - \hat{\theta}^{bs}_G$	0.330	0.721	0.390	0.515	0.101	0.521
$\hat{\theta}^{bs}_E - \hat{\theta}^{bs}_H$	0.455	0.816	0.360	0.619	0.092	0.611
$\hat{\theta}^{bs}_F - \hat{\theta}^{bs}_G$	1.376	1.732	0.355	1.555	0.094	1.567
$\hat{\theta}^{bs}_F - \hat{\theta}^{bs}_H$	1.502	1.832	0.330	1.659	0.085	1.657
$\hat{\theta}^{bs}_G - \hat{\theta}^{bs}_H$	-0.020	0.245	0.265	0.104	0.068	0.090

Table S5: Parametric Bootstrap CIs for $c = 40$; Significance (at level $\alpha = 0.05$) against $H_0 : \theta_i = \theta_j$ is in red; differences involving treatment G or H (which we are most interested in) are highlighted in yellow; assuming t-distributed noise.

	CI lower	CI upper	CI length	mean	sd	est
$\hat{\gamma}_A^{bs}$	4.194	5.000	0.806	4.491	0.183	4.424
$\hat{\gamma}_B^{bs}$	3.569	3.834	0.265	3.634	0.110	3.594
$\hat{\gamma}_C^{bs}$	3.634	4.274	0.641	3.882	0.185	3.859
$\hat{\gamma}_D^{bs}$	3.589	3.939	0.350	3.722	0.117	3.719
$\hat{\gamma}_E^{bs}$	4.089	5.000	0.911	4.761	0.325	5.000
$\hat{\gamma}_F^{bs}$	4.750	5.000	0.250	4.954	0.074	5.000
$\hat{\gamma}_G^{bs}$	3.539	3.899	0.360	3.647	0.162	3.599
$\hat{\gamma}_H^{bs}$	4.374	4.965	0.591	4.514	0.123	4.459
$\hat{\gamma}_A^{bs} - \hat{\gamma}_B^{bs}$	0.455	1.401	0.946	0.857	0.212	0.831
$\hat{\gamma}_A^{bs} - \hat{\gamma}_C^{bs}$	0.090	1.196	1.106	0.609	0.255	0.566
$\hat{\gamma}_A^{bs} - \hat{\gamma}_D^{bs}$	0.370	1.311	0.941	0.769	0.217	0.706
$\hat{\gamma}_A^{bs} - \hat{\gamma}_E^{bs}$	-0.771	0.551	1.321	-0.270	0.373	-0.576
$\hat{\gamma}_A^{bs} - \hat{\gamma}_F^{bs}$	-0.796	0.035	0.831	-0.463	0.198	-0.576
$\hat{\gamma}_A^{bs} - \hat{\gamma}_G^{bs}$	0.480	1.401	0.921	0.844	0.241	0.826
$\hat{\gamma}_A^{bs} - \hat{\gamma}_H^{bs}$	-0.501	0.526	1.026	-0.022	0.223	-0.035
$\hat{\gamma}_B^{bs} - \hat{\gamma}_C^{bs}$	-0.621	0.065	0.686	-0.248	0.217	-0.265
$\hat{\gamma}_B^{bs} - \hat{\gamma}_D^{bs}$	-0.335	0.150	0.485	-0.088	0.159	-0.125
$\hat{\gamma}_B^{bs} - \hat{\gamma}_E^{bs}$	-1.426	-0.395	1.031	-1.127	0.340	-1.406
$\hat{\gamma}_B^{bs} - \hat{\gamma}_F^{bs}$	-1.426	-1.076	0.350	-1.320	0.132	-1.406
$\hat{\gamma}_B^{bs} - \hat{\gamma}_G^{bs}$	-0.265	0.220	0.485	-0.013	0.196	-0.005
$\hat{\gamma}_B^{bs} - \hat{\gamma}_H^{bs}$	-1.336	-0.616	0.721	-0.880	0.169	-0.866
$\hat{\gamma}_C^{bs} - \hat{\gamma}_D^{bs}$	-0.165	0.541	0.706	0.160	0.217	0.140
$\hat{\gamma}_C^{bs} - \hat{\gamma}_E^{bs}$	-1.316	0.000	1.316	-0.879	0.374	-1.141
$\hat{\gamma}_C^{bs} - \hat{\gamma}_F^{bs}$	-1.346	-0.666	0.681	-1.072	0.198	-1.141
$\hat{\gamma}_C^{bs} - \hat{\gamma}_G^{bs}$	-0.145	0.666	0.811	0.235	0.247	0.260
$\hat{\gamma}_C^{bs} - \hat{\gamma}_H^{bs}$	-1.076	-0.225	0.851	-0.632	0.224	-0.601
$\hat{\gamma}_D^{bs} - \hat{\gamma}_E^{bs}$	-1.406	-0.305	1.101	-1.039	0.351	-1.281
$\hat{\gamma}_D^{bs} - \hat{\gamma}_F^{bs}$	-1.406	-0.951	0.455	-1.232	0.139	-1.281
$\hat{\gamma}_D^{bs} - \hat{\gamma}_G^{bs}$	-0.200	0.345	0.546	0.075	0.202	0.120
$\hat{\gamma}_D^{bs} - \hat{\gamma}_H^{bs}$	-1.206	-0.506	0.701	-0.792	0.170	-0.741
$\hat{\gamma}_E^{bs} - \hat{\gamma}_F^{bs}$	-0.861	0.225	1.086	-0.193	0.333	0.000
$\hat{\gamma}_E^{bs} - \hat{\gamma}_G^{bs}$	0.385	1.456	1.071	1.114	0.362	1.401
$\hat{\gamma}_E^{bs} - \hat{\gamma}_H^{bs}$	-0.495	0.596	1.091	0.248	0.348	0.541
$\hat{\gamma}_F^{bs} - \hat{\gamma}_G^{bs}$	1.006	1.456	0.450	1.307	0.176	1.401
$\hat{\gamma}_F^{bs} - \hat{\gamma}_H^{bs}$	0.000	0.606	0.606	0.440	0.141	0.541
$\hat{\gamma}_G^{bs} - \hat{\gamma}_H^{bs}$	-1.326	-0.551	0.776	-0.866	0.207	-0.861

Table S6: Parametric Bootstrap CIs for $c = 0.1$; Significance (at level $\alpha = 0.05$) against $H_0 : \gamma_i = \gamma_j$ is in red; differences involving treatment G or H (which we are most interested in) are highlighted in yellow; assuming t-distributed noise.
